# Supplemental *Clostridium butyricum* Modulates Lipid Metabolism Through Shaping Gut Microbiota and Bile Acid Profile of Aged Laying Hens

**DOI:** 10.3389/fmicb.2020.00600

**Published:** 2020-04-15

**Authors:** Wei-wei Wang, Jing Wang, Hai-jun Zhang, Shu-geng Wu, Guang-hai Qi

**Affiliations:** Risk Assessment Laboratory of Feed Derived Factors to Animal Product Quality Safety of Ministry of Agriculture & Rural Affairs, and National Engineering Research Center of Biological Feed, Feed Research Institute, Chinese Academy of Agricultural Sciences, Beijing, China

**Keywords:** *Clostridium butyricum*, laying hen, lipid metabolism, gut microbiota, bile acid

## Abstract

Probiotic *Clostridium butyricum* could affect lipid metabolism in broilers. However, it is not clear whether *C. butyricum* could improve lipid metabolism through shaping gut microbiota and bile acid (BA) profile of laying hens. We aimed to evaluate the contributions of gut microbiota and BA profile to the potential effect of *C. butyricum* on lipid metabolism of aged laying hens. A total of 192 60-week-old Hy-Line Brown laying hens were divided into two groups (eight replicates per group). Birds were fed a basal diet supplemented with 0 or 2.7 g/kg *C. butyricum* (1.0 × 10^9^ CFU/g). Samples were collected at the end of week 8 of the experiment. The results showed elevated (*P* < 0.05) concentrations of glucagon-like peptide 1, insulin and thyroid hormones in serum responded to *C. butyricum* addition, which also decreased (*P* < 0.05) hepatic free fatty acids contents, as well as increased (*P* < 0.05) the expression of hepatic acyl-CoA oxidase, farnesoid X receptor (FXR) and PPARα. *C. butyricum* addition increased (*P* < 0.05) *Bacteroidetes* abundance but tended to decrease (*P* < 0.10) *Firmicutes* abundance in the ileum. Besides, *C. butyricum* addition resulted in higher (*P* < 0.05) abundances of *Clostridia* (*Clostridiales*) and *Prevotellaceae*, concurrent with an increasing trend (*P* < 0.10) of *Bifidobacteriaceae* abundance and decreased the abundances of several harmful bacteria such as *Klebsiella* (*P* < 0.05). Regarding ileal BA profile, there was a reduced (*P* < 0.05) content of tauro-α-muricholic acid, increased (*P* < 0.05) contents of tauroursodeoxycholic acid and lithocholic acid, along with increasing trends (*P* < 0.10) of glycochenodeoxycholic acid and hyodeoxycholic acid contents due to *C. butyricum* addition, which also increased (*P* < 0.05) ileal FXR expression. Collectively, supplemental *C. butyricum* accelerated hepatic fatty acid oxidation, and shaped gut microbiota and BA profile, thus reducing fat deposition in the liver of aged laying hens.

## Introduction

Aged laying hens are known for their disturbance of lipid metabolism after having undergone the intensive metabolism at peak production (Boonsinchai, 2015; Liu et al., 2018), which could induce fat accumulation in the liver, thus leading to a defect of hepatic functionality of laying hens (Trott et al., 2014). Hence, there is a strong demand to regulate lipid metabolism of aged layers that could be critical for the practical production cost and animal welfare. Recent studies have focused on the importance of probiotics as a kind of regulator of lipid metabolism in chickens (Saleh et al., 2012, 2014). One of the potential mechanisms by which probiotics regulate lipid metabolism in chickens is through modifying the expression of lipogenesis-related genes such as acetyl-CoA carboxylase (ACC) and of lipolysis-related genes such as carnitine palmitoyltransferase in selected tissues such as muscle (Saleh et al., 2012, 2014). As an important constituent of probiotics, *Clostridium butyricum* principally colonizes in the distal small intestine and colon of animals (Sato and Tanaka, 1997). *C. butyricum* has been used to prevent or treat intestinal disorders of animals (Hayashi et al., 2013). Beyond the positive effects on intestinal tract, *C. butyricum* addition was reported recently to regulate lipid metabolism in various tissues of broiler chickens (Zhang et al., 2011; Zhao et al., 2013). However, no information is available regarding the effects of *C. butyricum* addition on lipid metabolism of laying hens.

Gut microbiota emerge as a crucial factor impacting the bioavailability of dietary components and exert a close connection with host nutritional and physiological processes, interacting with lipid metabolism and the development of obesity and fatty liver disorders of host (Lu et al., 2019; Monk et al., 2019; Zhao et al., 2019). There are obvious changes in gut microbial composition of laying hens with aging (Videnska et al., 2014). For aged hens, the gut microbiota dysbiosis was characterized by declined abundances of several beneficial bacteria (Videnska et al., 2014), which might be associated with the perturbation in their lipid metabolism (Jia et al., 2017). Previous studies have demonstrated that dietary *C. butyricum* could manipulate gut microbiota in chickens, as exhibited by the increased quantities of some beneficial bacteria and decreased quantities of certain harmful bacteria in broiler gut (Zhao et al., 2013; Zhang et al., 2014). Besides, the hepatoprotective roles of *C. butyricum* in mice have been suggested to be linked with its remodeling on gut microbial composition (Liu et al., 2017). Accordingly, *C. butyricum* addition might regulate lipid metabolism of chickens through shaping gut microbiota.

Microbial influence over bile acid (BA) processing represents an important mechanism through which gut microbiota exert impacts on host metabolism (Tang et al., 2019). BAs could serve as a kind of signaling molecules interacting with FXR that is highly expressed in enterohepatic tissues, influencing hepatic BA homeostasis and lipid metabolism of host through the interplay between gut and liver (Rajani and Jia, 2018; Tang et al., 2019). Since aging-related gut microbiota dysbiosis could facilitate the development of obesity and fatty liver disease presumably through modifications of the chemical and signaling properties of BAs (Fu et al., 2012; Carbajo-Pescador et al., 2019; Tang et al., 2019), there might be a deterioration of intestinal BA profile accompanied by gut microbiota dysbiosis in aged laying hens, thereby favoring the accumulation of fat in their livers. Previous researches have revealed alterations of BA contents in serum, liver and feces, concurrent with a shift in gut microbiota of host due to dietary *C. butyricum* (Sato et al., 2012; Seo et al., 2013), suggesting that *C. butyricum* treatment may elicit an alteration of intestinal BA composition. Nevertheless, there is a lack of understanding about the regulation of intestinal BA profile in chickens, by which gut microbiota manipulation could contribute to the potential modulation of lipid metabolism in laying hens as a result of *C. butyricum* addition.

Comprehensively, the present study was aimed at exploring if supplemental *C. butyricum* could be an approach for modulating lipid metabolism in aged laying hens by targeting intestinal microbiota and BA profile that mediate the gut-liver crosstalk.

## Materials and Methods

### Animals and Experimental Design

The experimental animal protocols for this study were approved by the Animal Care and Use Committee of the Feed Research Institute of the Chinese Academy of Agricultural Sciences (approval no. FRI-CAAS20190902). A total of 192 aged (60-week-old) Hy-Line Brown laying hens were divided into two groups with eight replicate cages (12 birds per replicate). These birds are being used in a separate study and that the birds used in the present study are just sub-samples. The initial body weight (control: 1.93 ± 0.15 kg; treatment: 1.91 ± 0.12 kg) and egg production (control: 87.50 ± 4.45%; treatment: 89.58 ± 3.86%) of birds were similar across all the replicates. All birds were acclimated to the diet and environment for 1 week. Afterward, birds were fed a basal diet without or with addition of 2.7 g/kg *C. butyricum* (1 × 10^9^ CFU/g) throughout the trial period. The additive dose of *C. butyricum* was based on our previous dose-response study (in review). The actual count (9.3 × 10^8^ CFU/g) of *C. butyricum* in this supplement was determined using spread plate method. The *C. butyricum* strain (HJCB998) was obtained from Huijia Biological Technology, Ltd. (Hangzhou, China). This supplement was firstly premixed with corn powder and then mixed in the compound feed. All birds were raised in three-tier battery cages and exposed to 16 h of light/d. Room temperature was maintained between 14 and 20°C. All birds were allowed free access to the water and mash feed. Composition of the basal diet based on the National Research Council (National Research Council [NRC], 1994) is shown in Table 1.

**TABLE 1:** Composition of the basal diet (air-dry basis).

**Ingredients**	**Content (%)**
Corn	64.52
Soybean meal (44% crude protein)	24.5
Limestone	8.9
Sodium chloride	0.3
Dicalcium phosphate	1.35
Choline chloride (50%)	0.1
DL-Methionine (98%)	0.1
Multimineral^1^	0.2
Multivitamin^2^	0.03
**Nutrient levels**	
Metabolizable energy (MJ/kg)	11.18
Crude protein (%)	16.16
Crude fat (%)	2.79
Total phosphorus (%)	0.54
Available phosphorus (%)	0.34
Calcium (%)	3.5
Lysine (%)	0.8
Methionine (%)	0.35

*^1^Supplied per kilogram of diet: Cu, 8 mg; Zn, 66 mg; Fe, 60 mg; Mn, 65 mg; Se, 0.3 mg; I, 1 mg. ^2^Supplied per kilogram of diet: vitamin A, 12,500 IU; vitamin D_3_, 4,125 IU; vitamin E, 15 IU; menadione, 2 mg; thiamin, 1 mg; riboflavin, 8.5 mg; pyridoxine, 8 mg; cobalamin, 5 mg; pantothenic acid, 50 mg; niacin, 32.5 mg; biotin, 2 mg; folic acid, 5 mg; choline, 500 mg.*

### Sample Collection

At the end of week 8 of the experiment, one bird was randomly selected from each replicate cage (*n* = 8) and individual blood were taken aseptically from the wing vein after starvation for 10 h. Serum samples were then obtained by centrifugation of blood at 3,000 rpm for 10 min at 4°C and stored at −20°C. These birds were slaughtered rapidly after blood collection, a little patch of the liver from each bird was immediately removed into RNAStore solution (TIANGEN Biotech., Co. Ltd., Beijing, China) and stored at −80°C for RNA extraction. The remainder liver sample was collected for determination of hepatic lipids contents. Afterward, the intestinal tract of each bird was separated and the mid-point of ileal segment was then removed into RNAStore solution (TIANGEN Biotech., Co. Ltd., Beijing, China) and stored at −80°C for RNA extraction. Besides, digesta sample in the ileum was collected and quick-froze by liquid nitrogen, followed by storage at −80°C.

### Biochemical Assay of Serum Samples

Serum triglyceride (TG), total cholesterol (TC), high density lipoprotein cholesterol (HDL-C), and low density lipoprotein cholesterol (LDL-C) concentrations were measured using a KHB400 Automatic Biochemical Analyzer (Kehua Bioengineering, Co. Ltd., Shanghai, China). The ratio of HDL-C level to LDL-C level was calculated. Free fatty acids (FFAs) were quantified by colorimetry according to the protocols of a commercial kit (Jiancheng Bioengineering Institute, Nanjing, China). The concentrations of lipid metabolism-related hormones including glucagon, insulin, glucagon-like peptide 1 (GLP-1), leptin, triiodothyronine (T_3_) and thyroxine (T_4_) in serum were determined by radioimmunoassay (Huaying Biotechnology, Co. Ltd., Beijing, China).

### Measurement of Hepatic Lipid Contents

Total lipid was extracted from the liver sample that was pre-weighed, according to the method described by Folch et al. (1957). The dried liver lipid was then weighed to calculate the content of hepatic total lipid, which was expressed as the percentage of the weight of dried liver lipid to the wet weight of liver sample. The dried lipid was then dissolved in 2 mL butanol for measuring TC and TG contents with a VersaMax microplate reader (Molecular Devices, San Jose, CA, United States) by using commercial kits (Jiancheng Bioengineering Institute, Nanjing, China). In addition, another set of the liver samples were homogenized with cold saline. After centrifugation at 3000 rpm for 10 min at 4°C, the resultant supernatants were collected and FFAs contents were assayed colorimetrically using a commercial kit (Jiancheng Bioengineering Institute, Nanjing, China).

### RNA Isolation and Real-Time PCR

Total RNA was extracted from the liver and ileum samples by using Trizol Reagent (TIANGEN Biotech., Co. Ltd., Beijing, China) following the manufacturer’s instructions. The relative mRNA expression was quantified as previously described (Wang et al., 2019). Primer sequences for the reference gene (reduced glyceraldehyde-phosphate dehydrogenase) and target genes including ACC, fatty acid synthetase (FAS), sterol-regulatory element-binding protein 1c (SREBP1c), liver X receptor α (LXRα), farnesoid X receptor (FXR), acyl-CoA oxidase (ACOX), long-chain acyl-CoA dehydrogenase (LCAD), peroxisome proliferator activated receptor α (PPARα) and PPARγ are exhibited in Supplementary Table 1. The results of relative expression of genes were calculated using the 2^–ΔΔ*Ct*^ method.

### High-Throughput Sequencing of Gut Microbiota

Bacterial genomic DNA was extracted from ileal digesta using NucleoSpin^®^ DNA Stool kit (MACHEREY-NAGEL Company, Germany). The quality of DNA samples were checked with gel electrophoresis. Bacterial 16S rDNA sequences spanning the variable regions V3–V4 were amplified according to the method described previously (Wang et al., 2019). The sequencing of the PCR products was performed on an Illumina HiSeq2500 PE250 platform (Illumina, San Diego, CA, United States). The sequencing results has been submitted to the Sequence Read Archive of the NCBI (Accession no. SAMN13610217). All the effective reads were clustered into operational taxonomic units (OTUs) based on a 97% sequence similarity. Classification of OTUs at various taxonomic levels were implemented by comparing sequences to the GreenGene database. Microbial α-diversity was analyzed using the MOTHUR v1.31.2 program (Schloss et al., 2009). The principal co-ordinates analysis (PCoA) and plot and non-metric multidimensional scaling (NMDS) were used to assess pairwise distances among samples (β-diversity). Linear discriminant analysis (LDA) combined effect size measurements (LEfSe) was applied to identify the relative richness (LDA > 2, *P* < 0.05) of bacteria between groups.

### Targeted Metabolome Analysis of Intestinal BAs

The ultra high performance liquid chromatography-mass spectrometry (UPLC/MS) method was modified to determine BAs contents in ileal digesta (Tang et al., 2019). Briefly, approximately 50 mg of each sample was deproteinized by adding 1000 μL of extract solvent (Vacetonitrile: Vmethanol: Vwater = 2: 2: 1, containing 0.1% formic acid and 50 nmol/L internal standard). The mixture was vortexed for 30 s, homogenized at 35 Hz for 4 min, and sonication for 5 min in ice-water bath. These steps were repeated for three times, followed by incubation at −20°C for 1 h. After centrifugation at 11,000 rpm for 15 min at 4°C, the resulting supernatant was applied to the UPLC/MS system. The BAs were quantified by a 1290 Infinity series UHPLC System (Agilent Technologies, United States) coupled with a Q Exactive^TM^ Focus MS (Thermo Fisher Scientific, United States). An ACQUITY UPLC BEH column (150 mm × 2.1 mm, 1.7 μm, Waters) heated to 45°C was used for chromatographic separation. A gradient system consisted of the Solvent A (0.1% acetic acid) and the Solvent B (acetonitrile) at a flow rate of 0.3 mL/min. The auto-sampler temperature was set at 4°C and the injection volume was 3 μL. The ion source parameter was set as follows: spray voltage = + 3500/−3100 V, sheath gas flow rate = 40 L/h, aux gas flow rate = 15 L/h, aux gas temperature = 350°C, and capillary temperature = 320°C. BA standards were obtained from Sigma-Aldrich, Co. Ltd. (St. Louis, MO, United States). The mix reference standards were prepared by dissolving each BA reference standard respectively in methanol.

### Statistical Analysis

Data are presented as mean ± standard deviation and analyzed by using SPSS 18.0 software. *t*-Tests were used to detect effects of dietary treatment on serum and hepatic biochemical indices, hepatic and intestinal genes expression, as well as intestinal BAs contents. Wilcoxon rank tests were used to detect the differences in the relative abundances of bacteria between groups. Significance was defined as *P* < 0.05 and 0.05 ≤ *P* < 0.10 was considered to be a tendency toward significance.

## Results

### Lipid and Hormone Concentrations in Serum

*Clostridium butyricum* addition did not significantly alter (*P* > 0.10) serum TC, HDL-C, LDL-C, and FFAs concentrations of laying hens (Table 2), but tended to reduce (*P* = 0.091) serum TG concentration. Birds consumed a diet containing *C. butyricum* had significant higher (*P* < 0.05) concentrations of GLP-1, insulin, T_3_ and T_4_ in serum as compared with birds received a control diet (Table 3).

**TABLE 2:** Effects of *Clostridium butyricum* treatment on serum lipid concentrations^1^ of laying hens.

	**TC (mmol/L)**	**TG (mmol/L)**	**HDL-C (mmol/L)**	**LDL-C (mmol/L)**	**H/L**	**FFAs (mmol/L)**
Control	3.09 ± 0.64	4.04 ± 0.68	1.14 ± 0.42	0.38 ± 0.15	3.23 ± 1.02	0.39 ± 0.11
Treatment	3.46 ± 0.53	3.47 ± 0.57	1.12 ± 0.29	0.31 ± 0.10	3.69 ± 0.63	0.33 ± 0.12
*P*-value	0.230	0.091	0.913	0.335	0.296	0.331

*^1^TC, total cholesterol; TG, triglyceride; HDL-C, high density lipoprotein cholesterol; LDL-C, low density lipoprotein cholesterol; H/L, ratio of HDL to LDL; FFAs, free fatty acids.*

**TABLE 3:** Effects of *Clostridium butyricum* treatment on serum hormones^1^ concentrations of laying hens.

	**GLP-1 (pmol/L)**	**Glucagon (pg/mL)**	**Insulin (μIU/mL)**	**Leptin (ng/mL)**	**T_3_ (ng/mL)**	**T_4_ (ng/mL)**
Control	21.06 ± 3.69^b^	61.77 ± 7.62	10.10 ± 1.45^b^	4.15 ± 0.63	0.94 ± 0.07^b^	15.41 ± 1.44^b^
Treatment	26.62 ± 5.99^a^	67.84 ± 7.68	12.38 ± 1.74^a^	4.57 ± 0.58	1.19 ± 0.15^a^	19.49 ± 1.71^a^
*P*-value	0.042	0.135	0.031	0.196	0.001	< 0.001

*^*a,b*^Values with different superscripts within the same column differ significantly (*P* < 0.05). ^1^GLP-1, glucagon-like peptide 1; T_3_, triiodothyronine; T_4_, tetraiodothyronine.*

### Hepatic Lipid Contents, and Gene Expression in the Liver and Intestine

**FIGURE 1:**
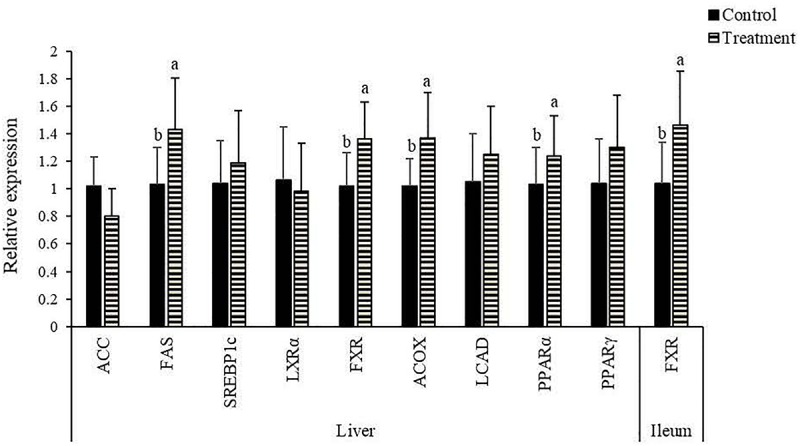
Effects of *Clostridium butyricum* treatment on the relative mRNA expression of lipid metabolism-related genes^1^ in the liver and ileum of laying hens. ^*a,b*^Treatments with unlike letters were significantly different (*P* < 0.05). ^1^ACC, acetyl-CoA carboxylase; FAS, fatty acid synthetase; SREBP, sterol-regulatory element-binding protein; LXR, liver X receptor; FXR, farnesoid X receptor; ACOX, acyl-CoA oxidase; LCAD, long-chain acyl-CoA dehydrogenase; PPAR, peroxisome proliferator activated receptor.

*Clostridium butyricum* treatment had no significant impacts (*P* > 0.10) on the contents of total lipid, TC and FFAs in the liver of birds (Table 4), but led to a decreasing trend (*P* = 0.062) of TG content with significantly reduced (*P* < 0.05) contents of FFAs in the liver. In terms of lipid metabolism-related genes, significantly increased (*P* < 0.05) mRNA expression of FAS, FXR, ACOX, and PPARα were observed in the liver of birds supplemented with *C. butyricum* (Figure 1), which also significantly up-regulated (*P* < 0.05) FXR expression in the ileum.

**TABLE 4:** Effects of *Clostridium butyricum* treatment on hepatic lipid contents^1^ of laying hens.

	**Total lipid content (%)**	**TC (μmol/g tissue)**	**TG (μmol/g tissue)**	**FFAs (μmol/g tissue)**
Control	13.57 ± 2.54	37.61 ± 5.58	90.75 ± 16.09	6.66 ± 1.17^a^
Treatment	16.08 ± 5.70	38.31 ± 6.76	74.34 ± 10.37	5.16 ± 1.35^b^
*P*-value	0.348	0.849	0.062	0.032

*^*a,b*^Values with different superscripts within the same column differ significantly (*P* < 0.05). ^1^Total lipid content was expressed as the percentage of the weight of dried liver lipid to the wet weight of liver sample. TC, total cholesterol; TG, triglycerides; FFAs, free fatty acids.*

### Diversity of Gut Microbiota

**FIGURE 2:**
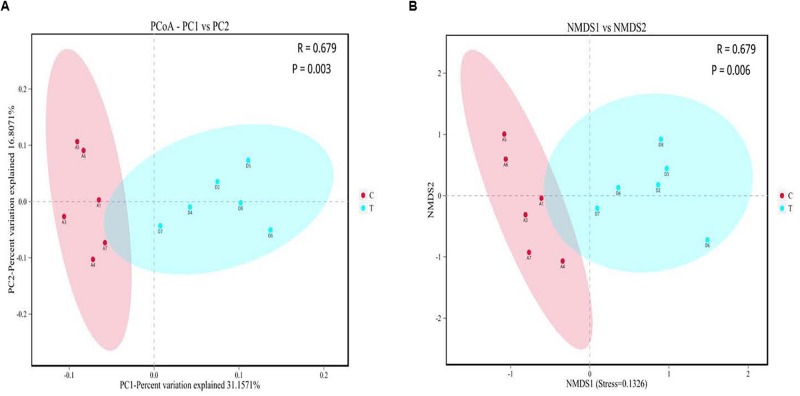
Beta diversity analysis of gut microbiota between the control and *Clostridium butyricum* treatment groups. **(A)** Principal co-ordinates analysis (PCoA) plot; **(B)** Non-metric multidimensional scaling (NMDS) plot. C, control group; T, *C. butyricum* treatment group.

No significant changes (*P* > 0.10) occurred in the α-diversity including abundance-based coverage estimator (ACE) and Chao1 estimator along with Shannon and Simpson indexes of ileal microbiota of birds in response to *C. butyricum* addition (Supplementary Table 2). There were significant separations (*P* < 0.05) of ileal microbial communities between groups, as visualized by both PCoA and NMDS plots (Figure 2).

### Composition of Gut Microbiota

The dominated phylum in the ileum of birds was *Firmicutes*, which contributed greater than 95% of the whole phyla (Supplementary Figure 1A). Other phyla were presented at very low relative abundances. The dominated class in the microbiota was *Bacilli*, followed by *Actinobacteria* (Supplementary Figure 1B). Within *Bacilli*, the majority belonged to the order *Lactobacillales* (mainly comprised the family *Lactobacteriaceae*), while the main order within *Actinobacteria* was *Bifidobacteriales* (Supplementary Figure 1C). Family level analysis showed that the microbiota was dominated by *Lactobacillaceae*, followed by *Bifidobacteriaceae* (Supplementary Figure 1D). At genus level, the *Lactobacillus* followed by *Aeriscardovia* accounted for the largest proportion of the microbiota (Supplementary Figure 1E).

**FIGURE 3:**
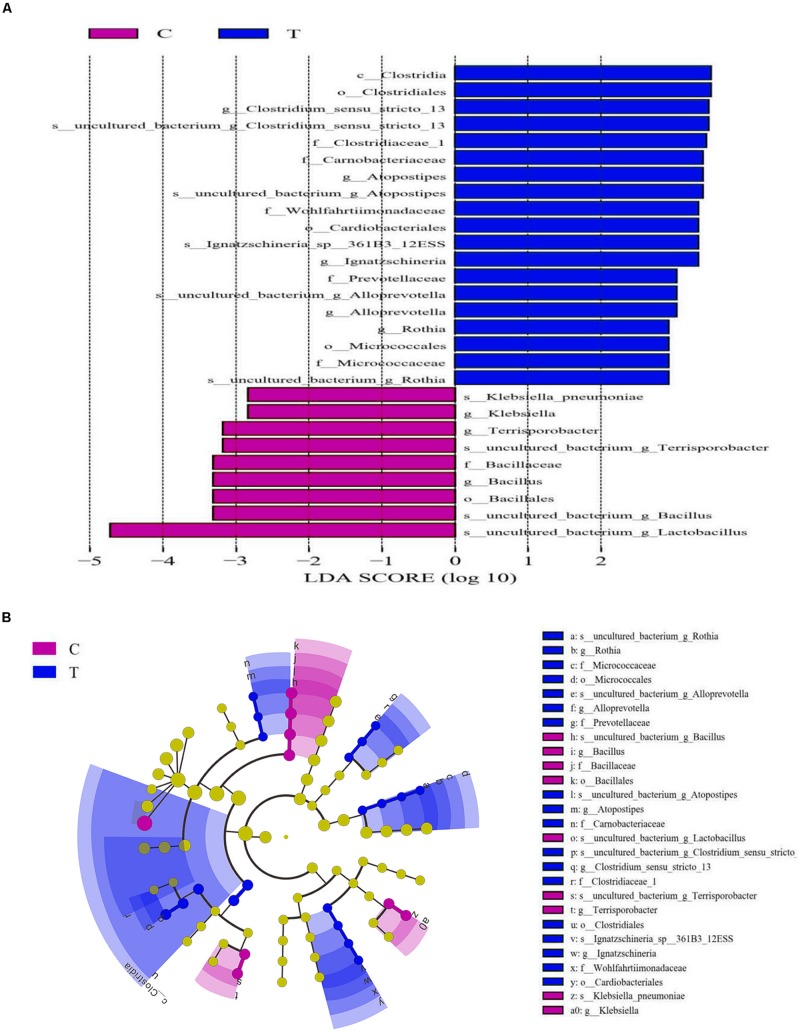
Linear discriminant analysis (LDA) combined effect size measurements (LEfSe) analysis of ileal microbiota of laying hens. **(A)** Species with significant differences (*P* < 0.05) that have an LDA score greater than the estimated value (2.0). **(B)** The cladogram diagram shows the microbial species with significant differences between groups. The species classification at the levels of phylum, class, order, family, and genus exhibited from the inside to the outside. C, control group; T, *Clostridium butyricum* treatment group.

The Wilcoxon rank tests identified a number of bacteria at various taxonomic levels as biomarkers to distinguish groups (Table 5). Treating birds with *C. butyricum* significantly elevated (*P* < 0.05) the abundances of *Bacteroidetes* and *Clostridia* (*Clostridiales*), and tended to increase (*P* = 0.061) *Bacteroidia* abundance, as well as tended to reduce (*P* < 0.10) the abundances of *Firmicutes* and *Bacilli*. *C. butyricum* treatment also significantly lowered (*P* < 0.05) the abundances of *Bacillaceae* (*Bacillus*) and tended to reduce (*P* = 0.088) *Lactobacillales* abundance, but showed a tendency to increase (*P* = 0.078) the abundances of *Bifidobacteriales* (*Bifidobacteriaceae*). There was a decreasing trend (*P* = 0.068) of *Enterobacteriaceae* abundance with a significant reduction (*P* < 0.05) of *Klebsiella* abundance in treatment group compared with control. Moreover, *C. butyricum* treatment induced significant elevations (*P* < 0.05) in the abundances of *Corynebacteriaceae* (*Corynebacterium*), *Psychrobacter* and *Rothia*, along with increasing trends (*P* < 0.10) of the abundances of *Brevibacteriaceae* (*Brevibacterium*). LEfSe analysis revealed enrichments (LDA > 2, *P* < 0.05) of several other beneficial bacteria such as the family *Prevotellaceae* and the genus *Alloprevotella* in the ileum of treatment group (Figure 3). Remarkably, the microbiota from treatment group was also enriched (LDA > 2, *P* < 0.05) with the genus *Clostridium sensu stricto* 13, which is probably derived from the increase of species *C. butyricum* in the ileum.

**TABLE 5:** Differences of bacterial distribution (%) in ileal digesta between the control and *Clostridium butyricum* treatment groups.

	**Control**	**Treatment**	***P*-value**
**Phyla**			
*Bacteroidetes*	0.130.04^b^	0.200.09^a^	0.044
*Firmicutes*	98.700.84	95.064.87	0.061
**Classes**			
*Clostridia*	0.350.13^b^	0.920.52^a^	0.011
*Bacilli*	98.300.90	94.105.14	0.055
*Bacteroidia*	0.130.04	0.200.09	0.061
**Orders**			
*Cardiobacteriales*	0.0020.002^b^	0.0510.010^a^	<0.001
*Bacillales*	0.3950.160^a^	0.0110.006^b^	<0.001
*Micrococcales*	0.0210.009^b^	0.0660.031^a^	0.004
*Clostridiales*	0.3490.126^b^	0.9160.516^a^	0.018
*Enterobacteriales*	0.0820.042	0.0480.019	0.059
*Bifidobacteriales*	0.5100.646	2.4741.162	0.078
*Lactobacillales*	97.940.99	94.125.14	0.088
**Families**			
*Wohlfahrtiimonadaceae*	0.0020.002^b^	0.0510.010^a^	<0.001
*Bacillaceae*	0.3950.160^a^	0.0100.007^b^	<0.001
*Corynebacteriaceae*	0.0140.007^b^	0.0270.007^a^	0.012
*Micrococcaceae*	0.0090.003^b^	0.0490.031^a^	0.012
*Carnobacteriaceae*	0.0170.009^b^	0.0340.014^a^	0.030
*Clostridiaceae*	0.1660.072	0.6290.548	0.060
*Enterobacteriaceae*	0.0820.042	0.0480.019	0.068
*Brevibacteriaceae*	0.0010.002	0.0020.002	0.072
*Bifidobacteriaceae*	0.5100.646	2.4741.162	0.078
**Genera**			
*Ignatzschineria*	0.0020.002^b^	0.0510.010^a^	<0.001
*Bacillus*	0.3950.160^a^	0.0100.007^b^	<0.001
*Brachybacterium*	0.0010.002^b^	0.0100.003^a^	<0.001
*Psychrobacter*	0.0010.001^b^	0.0080.003^a^	0.001
*Corynebacterium*	0.0080.006^b^	0.0170.005^a^	0.011
*Rothia*	0.0080.002^b^	0.0430.028^a^	0.013
*Klebsiella*	0.0360.026^a^	0.0060.004^b^	0.013
*Terrisporobacter*	0.0550.022^a^	0.0310.010^b^	0.025
*Clostridium sensu stricto 13*	0.0080.006	0.5220.575	0.054
*Brevibacterium*	0.0010.002	0.0020.002	0.079

*^*a,b*^Values with different superscripts within the same row differ significantly (*P* < 0.05).*

### Intestinal BA Profile

**FIGURE 4:**
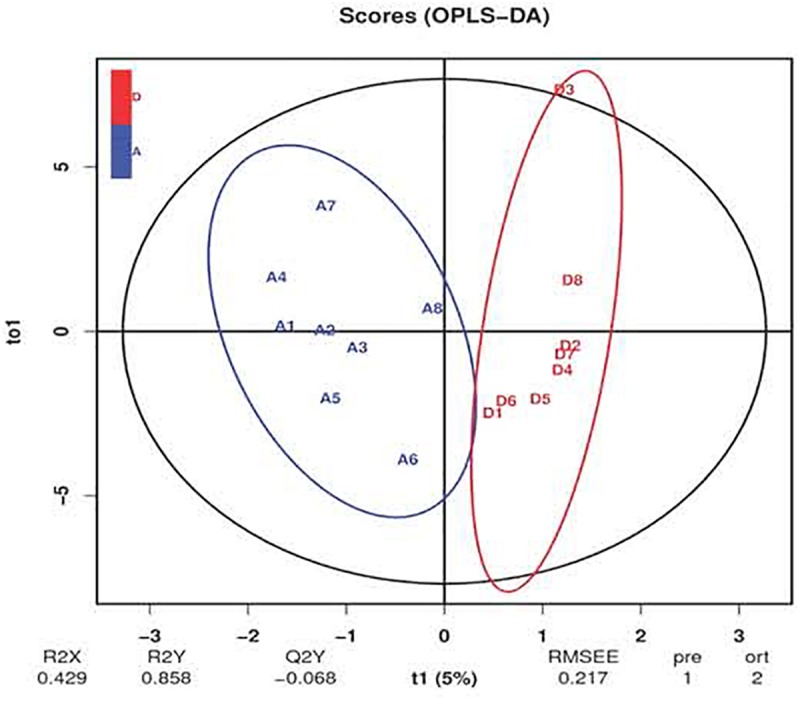
Orthogonal partial least squares discrimination analysis (OPLS-DA) of ileal bile acid profile of laying hens. The blue letters represent the samples of control group, while the red letters represent the samples from *Clostridium butyricum* treatment group.

The ion recording chromatographs showed excellent peak shapes with good separations among BA standard solutions (Supplementary Figure 2). Orthogonal partial least squares discrimination analysis (OPLS-DA) revealed an obvious separation of ileal BA profile between groups (Figure 4). Chenodeoxycholic acid, taurochenodeoxycholic acid, and cholic acid accounted for the largest proportion of primary BAs in the ileum of birds (Table 6), while allocholic acid, λ-muricholic acid, and 7-ketolithocholic acid represented the major secondary BAs in the ileum. There was a significant reduced (*P* < 0.05) content of tauro-α-muricholic acid (TαMCA) with a decreasing trend (*P* = 0.065) of taurohyodeoxycholic acid content in the ileum due to *C. butyricum* treatment, which also significantly elevated (*P* < 0.05) the contents of tauroursodeoxycholic acid (TUDCA), lithocholic acid (LCA) and 7-ketolithocholic acid, as well as tended to increase (*P* < 0.10) the contents of glycochenodeoxycholic acid (GCDCA) and hyodeoxycholic acid (HDCA) in the ileum.

**TABLE 6:** Effects of *Clostridium butyricum* treatment on ileal bile acids (BAs)^1^ contents of laying hens.

	**Control**	**Treatment**	***P*-value**
**Primary BAs (nmol/g)**			
TαMCA	31.0111.88^a^	17.586.24^b^	0.015
TβMCA	3.681.58	2.961.64	0.426
GCA	0.730.43	1.070.47	0.153
αMCA	5.244.64	5.443.66	0.926
βMCA	0.390.12	0.380.20	0.933
CDCA	4520.76821.24	4836.781022.99	0.456
CA	496.84215.04	498.89255.05	0.986
GCDCA	4.331.73	6.652.90	0.072
TCDCA	3065.881571.45	3923.872476.42	0.454
**Secondary BAs (nmol/g)**			
TUDCA	0.690.44^b^	2.672.52^a^	0.046
THDCA	1.210.84	0.570.31	0.065
UCA	0.660.34	0.700.62	0.885
UDCA	5.001.81	4.362.48	0.555
7-KDCA	0.390.16	0.660.45	0.130
12-DHCA	3.610.99	4.112.16	0.568
λMCA	57.0342.35	45.7124.76	0.553
3-DHCA	1.270.52	1.880.92	0.122
LCA	0.310.12^b^	0.650.33^a^	0.016
7-KLCA	9.852.09^b^	17.467.32^a^	0.009
12-KLCA	0.310.16	0.400.15	0.273
DCA	0.560.22	0.580.13	0.763
ApCA	9.603.21	8.543.33	0.502
AlCA	127.8239.25	114.4142.87	0.499
HDCA	0.810.37	1.170.37	0.056
TLCA	0.670.40	0.860.38	0.366

*^*a,b*^Values with different superscripts within the same row differ significantly (*P* < 0.05). ^1^TαMCA, tauro-α-muricholic acid; TβMCA, tauro-β-muricholic acid; GCA, glycocholic acid; αMCA, α-muricholic acid; βMCA, β-muricholic acid; CDCA, chenodeoxycholic acid; CA, cholic acid; GCDCA, glycochenodeoxycholic acid; TCDCA, taurochenodeoxycholic acid; TUDCA, tauroursodeoxycholic acid; THDCA, taurohyodeoxycholic acid; UCA, ursocholic acid; UDCA, ursodeoxycholic acid; 7-KDCA, 7-ketodeoxycholic acid; 12-DHCA, 12-dehydrocholic acid; λMCA, λ-muricholic acid; 3-DHCA, 3-dehydrocholic acid; LCA, lithocholic acid; 7-KLCA, 7-ketolithocholic acid; 12-KLCA, 12-ketolithocholic acid; DCA, deoxycholic acid; ApCA, apocholic acid; AlCA, allocholic acid; HDCA, hyodeoxycholic acid; TLCA, taurolithocholic acid.*

## Discussion

Serum lipid and lipoprotein concentrations are indicative of the basal adjustment of fatty acid (FA) circulation between the liver and peripheral tissues (Mossab et al., 2002). LDL is the main carrier of circulating FAs and cholesterol from the liver to extrahepatic tissues, and LDL-C serves as a risk factor for atherosclerosis (Jacobson et al., 2012). Contrastively, HDL carries FAs and cholesterol from peripheral tissues to the liver for catabolism, and HDL-C functions as an indicator of excretion of the surplus cholesterol to gut (Jacobson et al., 2012). Accumulating studies have indicated that *C. butyricum* addition tended to decrease serum TG and TC contents, but failed to modify serum lipoprotein cholesterol level in broilers (Zhang et al., 2011; Zhao et al., 2013). Similarly, the present study showed that administration of *C. butyricum* into layer diet resulted in a decreasing trend of TG level without an overall alteration of lipid profile in serum. It is well-known that lipid metabolism is strongly regulated by a variety of hormones, in which glucagon and insulin have negative and positive regulations on lipogenesis, respectively (Li et al., 2019). Leptin that produced by adipose tissue is related to the negative regulation of lipid deposition (Li et al., 2019). Thyroid hormones such as T_3_ and T_4_ are the main hormones involved in the catabolic processes of lipids (Li et al., 2019). GLP-1 is an intestinal signal peptide hormone that capable of regulating glucose-dependent secretion of insulin, but also elicits a promotion of lipid oxidation (Mulvihill, 2018). It was reported that *C. butyricum* addition increased serum insulin level of broilers (Zhao et al., 2013), and promoted GLP-1 secretion in mice (Jia et al., 2017). Herein, *C. butyricum* addition elevated serum insulin level, together with GLP-1, T_3_ and T_4_ levels, implying a promotion of lipogenesis with a simultaneous enhancement of lipolysis in laying hens due to *C. butyricum* addition. Alternatively, the increased levels of GLP-1, T_3_ and T_4_ following *C. butyricum* addition might represent an adaptive response of the corresponding elevation of insulin (Li et al., 2019).

Probiotic strains have been suggested to elicit lipid-lowering effect via several underlying mechanisms such as modulating lipid metabolism-related gene expression and enzyme activities (Yoo et al., 2013). In the present study, the reduction of hepatic FFA content with the decreasing trend of hepatic TG content suggested a reduced lipid deposition in the liver of layers due to *C. butyricum* addition, which might cause a decreased risk of fatty liver syndrome (Trott et al., 2014). Lipid deposition inside the body is largely dependent on the balance between lipolysis and lipogenesis. Among genes involved in lipogenic processes, ACC is the first rate-limiting enzyme of *de novo* synthesis of FAs, FAS also functions as a key determinant for the maximal capacity of lipogenesis, due to its role in catalyzing the elongation of carbon chains of FAs (Lu et al., 2018). In this study, hepatic FAS expression was increased in treatment group, which might result in a greater capacity of lipogenesis. This was in accordance with a previous study in broilers (Zhao et al., 2013). Since insulin secretion can activate the transcription of FAS by binding to its promoter (Griffin and Sul, 2004), the increased expression of FAS following *C. butyricum* addition was likely related to the elevated level of insulin. As a nuclear transcription factor binding to the sterol-regulatory element, SREBP1c facilitates biosynthesis of FAs and their incorporation into TG by directly promoting the expression of its target genes such as ACC and FAS (Horton et al., 2002). LXRα is a ligand-activated nuclear receptor with important functions in transcriptional control of FA synthesis by promoting SREBP1c expression and activating the promoters of ACC and FAS (Wang and Tontonoz, 2018). PPARs are molecular sensors of FAs and their derivatives. Among them, PPARγ is involved in lipogenic process by promoting the transcription of its target gene SREBP1c (Mirza and Sharma, 2019). In this study, no differences were detected in hepatic PPARγ, LXRα and SREBP1c expression between groups, suggesting that *C. butyricum*-induced up-regulation of FAS expression was not through the pathway of PPARγ–LXRα–SREBP1c. As the rate-limiting enzymes in lipolytic processes, LCAD and ACOX are respectively associated with catalyzation of the initial steps of mitochondrial and peroxisomal FA β-oxidation (Chu et al., 1994; Knottnerus et al., 2018). ACOX is characterized by a PPAR response element and responds to a change in FA level in a PPARα-dependent manner (Ringseis and Eder, 2009; Knottnerus et al., 2018). FXR acts as a receptor of BAs, playing critical roles in regulating BA homeostasis and lipid metabolism (Fang et al., 2015; Rajani and Jia, 2018). A targeted disruption of FXR could induce lipid accumulation in both serum and liver (Sinal et al., 2000). In contrast, FXR activation could reduce hepatic lipids levels through the positive regulation of PPARα, highlighting a role of FXR in accelerating lipolysis (Rajani and Jia, 2018). Thus, the increased expression of hepatic ACOX, PPARα and FXR in treatment group suggested that *C. butyricum* addition stimulated the peroxisomal FA β-oxidation probably through FXR–PPARα–ACOX pathway, thereby accounting for the corresponding reduced contents of hepatic FFAs and TG. Similarly, Weng et al. (2015) reported that *C. butyricum* addition promoted the peroxisomal β-oxidation of hepatic FAs via PPARα–ACOX pathway in mice. Seo et al. (2013) also observed an up-regulation of hepatic PPARα expression due to *C. butyricum* addition, possibly benefiting the inhibition of lipid accumulation in rats.

Gut microbiota exert essential roles in contributing to the modulation of host lipid metabolism by dietary intervention (Lu et al., 2019; Ushiroda et al., 2019). It was found that obese animals consistently showed a decrease in *Bacterioidetes* and an increase in *Firmicutes* in gut (Lu et al., 2019). More studies have validated that the increase of *Firmicutes* with the reduction of *Bacteroidetes* in gut exert a strong linkage with host lipid accumulation and fatty liver (Fontaine et al., 2019; Ushiroda et al., 2019). Consequently, the increased abundance of *Bacteroidetes* with the decreasing trend of *Firmicutes* abundance could be at least partially responsible for the reduced lipid deposition in the liver of layers as a result of *C. butyricum* addition. The increase of *Bacilli* and the reduction of *Bacteroidia* in gut were implicated in high-fat diet-induced obesity of animals (Fontaine et al., 2019; Ushiroda et al., 2019). *Clostridia* represents a critical class associated with short-chain fatty acids production that favors the inhibition of lipid accumulation and obesity (Murugesan et al., 2018). Within *Clostridia*, the order *Clostridiales* and the family *Clostridiaceae* were evidenced to be decreased in gut due to obesity (Monk et al., 2019; Zhao et al., 2019). In contrast, an increased abundance of *Lactobacillales* in gut was associated with obesity of animals (Fontaine et al., 2019; Lu et al., 2019). In this study, *C. butyricum* addition increased the abundances of *Clostridia* (including *Clostridiales* and *Clostridiaceae*) and *Bacteroidia*, as well as decreased *Lactobacillales* abundance and tended to reduce *Bacilli* abundance, which could be beneficial for decreasing lipid deposition in the liver of layers. *Enterobacteriaceae* especially the genus *Klebsiella* were identified as harmful bacteria with massive production of lipopolysaccharide (Keskitalo et al., 2018; Pope et al., 2019), which could trigger systemic inflammation and metabolic endotoxemia, contributing to the defective hepatic functions and lipid accumulation (Cani et al., 2007). As a well-known probiotic, *Bifidobacterium* was found to be reduced in the gut of obese animals (Wang et al., 2015). A previous study has confirmed the capability of *Bifidobacterium* treatment to suppress lipid accumulation and reduce the risk of obesity probably through attenuating endotoxemia (Wang et al., 2015). Herein, *C. butyricum* addition tended to elevate *Bifidobacteriales* (*Bifidobacteriaceae*) abundance but reduced *Enterobacteriales* and *Klebsiella* abundances, which might lower the susceptibility of lipid deposition and thus favor the reduction of lipid deposition in the liver. Similarly, Shang et al. (2016) reported that feeding *C. butyricum* attenuated endotoxemia of mice, through which *C. butyricum* inhibit lipid accumulation in the liver. As a producer of certain functional substances such as succinate, *Prevotellaceae* exerts a negative relationship with lipid deposition in animals (Zhang et al., 2019). A further study revealed reduced abundances of *Prevotellaceae* (*Prevotella*) and *Alloprevotella* in gut of animals with hepatic steatosis (Tang et al., 2019). Thereby, the enrichment of *Prevotellaceae* and *Alloprevotella* in *C. butyricum* treatment group might also favor the reduction of lipid deposition in the liver of laying hens.

BAs are well-known to possess metabolic functions in cholesterol homeostasis and lipid metabolism. The shift of BA profile induced by gut microbiota is closely associated with hepatic lipid accumulation of host (Tang et al., 2019). *C. butyricum* addition was reported to modify BAs contents in serum, liver and feces, as well as manipulate gut microbiota (Sato et al., 2012; Seo et al., 2013), suggesting that *C. butyricum* addition may shape intestinal BA profile of host. Indeed, the current study revealed that layers fed with *C. butyricum* had an alteration of ileal BA profile, as characterized by a reduced content of TαMCA, increased contents of TUDCA and LCA, together with increasing trends of GCDCA and HDCA contents. Several endogenous BAs can function as signaling molecules to interact with FXR, regulating not only their own synthesis and enterohepatic recirculation but also lipid metabolism (Rajani and Jia, 2018). Thereinto, TαMCA was identified as a FXR antagonist, whose reduction led to an elevated activity of FXR (Sayin et al., 2013). Conversely, LCA, GCDCA, TUDCA and HDCA were recognized as FXR agonists (Makishima et al., 2002; Marquardt et al., 2017; Wang et al., 2018). Remarkably, LCA and HDCA were found to be decreased in serum or intestine of animals with hepatic steatosis (Tang et al., 2019; Ushiroda et al., 2019), while dietary HDCA could exert hypolipidemic effects by reducing FXR antagonist BAs in enterohepatic tissues (Watanabe and Fujita, 2014). Accordingly, the current increases in TUDCA and LCA contents with the increasing trends of GCDCA and HDCA contents, as well as the reduced TαMCA content in treatment group were naturally answerable to the corresponding elevation of ileal FXR expression. This could subsequently stimulate PPARα-dependent β-oxidation of hepatic FAs through gut-liver crosstalk (Rajani and Jia, 2018), presumptively contributing to the reduced lipid deposition in the liver following *C. butyricum* addition. Because the majority of BAs are reabsorbed in the ileum into portal blood and re-circulated back to the liver for uptake by hepatocytes (Rajani and Jia, 2018; Tang et al., 2019), the elevated LCA, TUDCA, GCDCA and HDCA in the ileum induced by *C. butyricum* might transport to the liver via enterohepatic circulation and then directly activated hepatic FXR (as supported by the elevated expression of hepatic FXR in treatment group), thereby resulting in a repression of lipid deposition in the liver.

Biotransformation of BAs by gut microbiota through defined enzymatic activities such as deconjugation and dihydroxylation is a critical modulator of BA profile (Rajani and Jia, 2018; Ovadia et al., 2019). Associations between gut microbiota and the contents of various BAs have been previously confirmed (Tang et al., 2019; Ushiroda et al., 2019). Ovadia et al. (2019) observed a reduced conjugation of BAs in gut due to the increase of *Bacteroidetes*. Tang et al. (2019) indicated that *Bacteroidetes* favors HDCA formation probably by promoting deconjugation and dihydroxylation of primary BAs. Moreover, the abundances of *Bacteroidetes* together with *Prevotellaceae* were evidenced to be positively correlated with HDCA content, which might be the reason why their reductions contributes to obesity of host (Tang et al., 2019). Ushiroda et al. (2019) detected a positive correlation between *Prevotellaceae* abundance and LCA content. Herein, we found elevations in ileal LCA and HDCA contents concomitant with increased abundances of *Bacteroidetes* and *Prevotellaceae* in treatment group, suggesting that *C. butyricum* addition might promote LCA and HDCA production of layers by enriching *Bacteroidetes* and *Prevotellaceae* in gut. *Clostridiales* represents a typical producer of bile salt hydrolase, which catalyzes the deconjugation of conjugated BAs and promotes the formation of secondary BAs (Parasar et al., 2019). A positive relationship was identified between *Clostridiales* abundance in gut and deconjugation of taurocholic acid in animals (Ushiroda et al., 2019). Consequently, the increased abundance of *Clostridiales* following *C. butyricum* addition might promote the deconjugation of TαMCA, causing a corresponding decrease in ileal TαMCA content.

## Conclusion

Supplemental *C. butyricum* accelerated FA oxidation and reduced lipid deposition in the liver of aged laying hens, which could be associated to the capability to shape gut microbial composition and BA profile that mediate the gut-liver crosstalk. The current study can expand our fundamental knowledge concerning the roles of gut microbiota in mediating the regulatory effect of *C. butyricum* on lipid metabolism, which certainly helps to improve the efficiency in applying *C. butyricum* in chicken production.

## Data Availability Statement

The datasets generated for this study can be found in the Sequence Read Archive of the NCBI (Accession No. SAMN13610217).

## Ethics Statement

The animal study was reviewed and approved by the Animal Care and Use Committee of the Feed Research Institute of the Chinese Academy of Agricultural Sciences.

## Author Contributions

WW performed the experiment and wrote the manuscript. JW and HZ assisted with data analysis. SW and GQ contributed to the experimental design and preparation of the manuscript. All authors discussed the results and reviewed the manuscript and read and approved the final manuscript.

## Conflict of Interest

The authors declare that the research was conducted in the absence of any commercial or financial relationships that could be construed as a potential conflict of interest.

## Abbreviations

ACC: acetyl-CoA carboxylase
BA: bile acid
FAS: fatty acid synthetase
FFA: free fatty acid
FXR: farnesoid X receptor
GCDCA: glycochenodeoxycholic acid
GLP: glucagon-like peptide
HDCA: hyodeoxycholic acid
HDL-C: high-density lipoprotein cholesterol
LCA: lithocholic acid
LCAD: long-chain acyl-CoA dehydrogenase
LDA: linear discriminant analysis
LDL-C: low-density lipoprotein cholesterol
LXR,: liver X receptor
FXR: acyl-CoA oxidase (ACOX)
NMDS: non-metric multidimensional scaling
PCoA: principal coordinates analysis
PPAR: peroxisome proliferator activated receptor
SREBP: sterol-regulatory element-binding protein
T_3_: triiodothyronine
T_4_: thyroxine
T α MCA: tauro- α-muricholic acid
TC: total cholesterol
TG: triglyceride
TUDCA: tauroursodeoxycholic acid.
