# Supplementary material for: Supplemental Clostridium butyricum Modulates Lipid Metabolism Through Shaping Gut Microbiota and Bile Acid Profile of Aged Laying Hens
Source: Front Microbiol. 2020 Apr 15;11:600. doi: 10.3389/fmicb.2020.00600 (PMC7176355; doi:10.3389/fmicb.2020.00600)
Supplement: Supplementary file 1 [file Data_Sheet_1.docx]

**Supplementary Table 1.** Sequences for real-time PCR primers

| Genes^1^ |  | Primer sequence^2^ (5′-3′) | Accession no. |
| --- | --- | --- | --- |
| GAPDH |  | F:TGCTGCCCAGAACATCATCC | NM_204305 |
|  |  | R:ACGGCAGGTCAGGTCAACAA |  |
| ACC |  | F:AATGGCAGCTTTGGAGGTGT | NM205505 |
|  |  | R:TCTGTTTGGGTGGGAGGTG |  |
| FAS |  | F:CCAACGATTACCCGTCTCAA | J03860 |
|  |  | R:CAGGCTCTGTATGCTGTCCAA |  |
| SREBP1c |  | F:GCCCTCTGTGCCTTTGTCTTC | AY029224 |
|  |  | R:ACTCAGCCATGATGCTTCTTCC |  |
| LXRα |  | F:CAAAGGGAATGAATGAGC | AF492498 |
|  |  | R:AGCCGAAGGGCAAACAC |  |
| FXR |  | F:AGTAGAAGCCATGTTCCTCCGTT | AF492497 |
|  |  | R:GCAGTGCATATTCCTCCTGTGTC |  |
| ACOX |  | F:ATGTCACGTTCACCCCATCC | NM001006205 |
|  |  | R:AGGTAGGAGACCATGCCAGT |  |
| LCAD |  | F:CGTGGTGATTGTGGTTACGGTTA | NM_001006511 |
|  |  | R:TGTTCTCTTTCCCAAGCAAGGC |  |
| PPARα |  | F:TCCTTCCCGCTGACCAAA | AF163809 |
|  |  | R:TCCTGCACTGCCTCCACA |  |
| PPARγ |  | F:CGAGGAGTCTTCCAACTC | AF163811 |
|  |  | R:CCTGATGGCATTATGTGA |  |

^1^ GAPDH, reduced glyceraldehyde-phosphate dehydrogenase; ACC, acetyl-CoA carboxylase; FAS, fatty acid synthetase; SREBP, sterol-regulatory element-binding protein; LXR, the liver X receptor; FXR, farnesoid X receptor; ACOX, acyl-CoA oxidase; LCAD, long-chain acyl-CoA dehydrogenase; PPAR, peroxisome proliferator activated receptor.

^2^ F, forward; R, reverse.

**Supplementary Table 2.** Alpha diversity of ileal microbiota of aged laying hens

|  | ACE estimator | Chao1 estimator | Simpson index | Shannon index |
| --- | --- | --- | --- | --- |
| Control | 82.35±3.88 | 82.99±5.57 | 0.24±0.02 | 1.87±0.07 |
| Treatment | 88.96±9.99 | 89.34±11.11 | 0.20±0.06 | 2.02±0.22 |
| *P*-value | 0.161 | 0.239 | 0.222 | 0.145 |

| A. At phylum level | B. At class level |
| --- | --- |
| 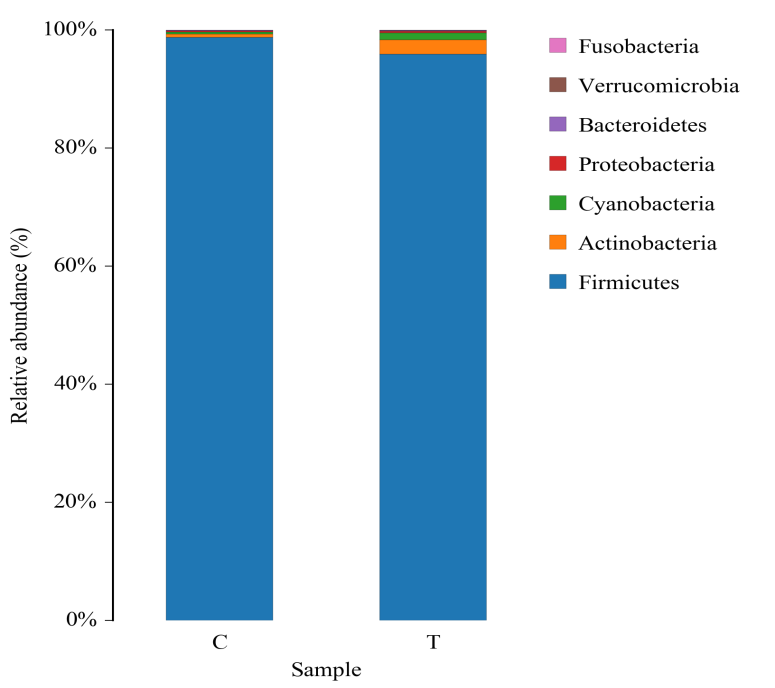 | 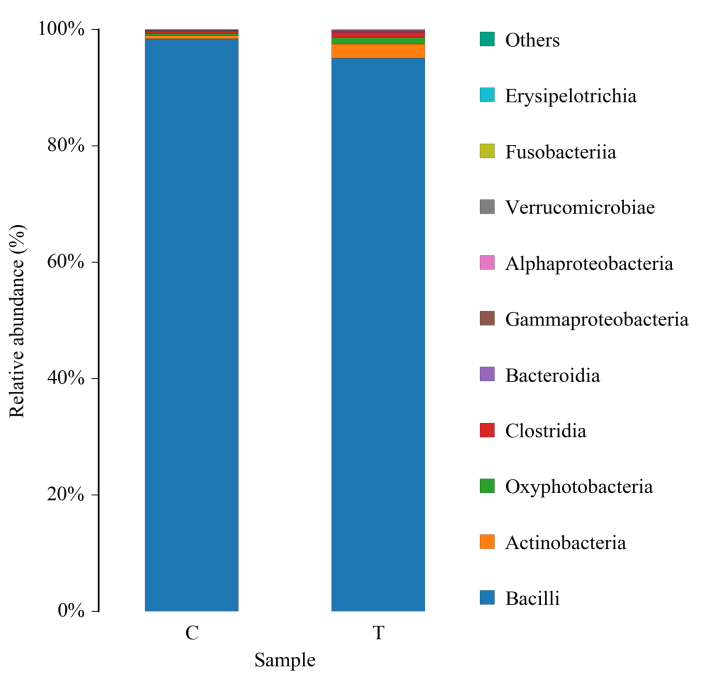 |
| C. At order level | D. At family level |
| 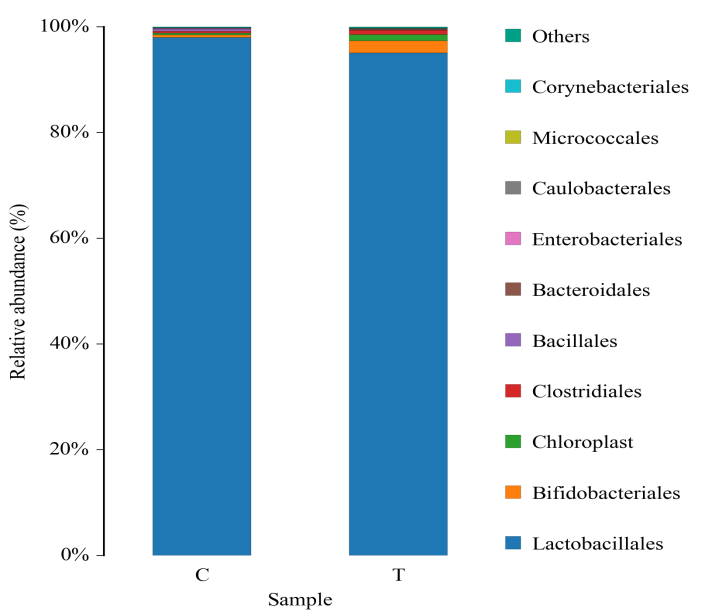 | 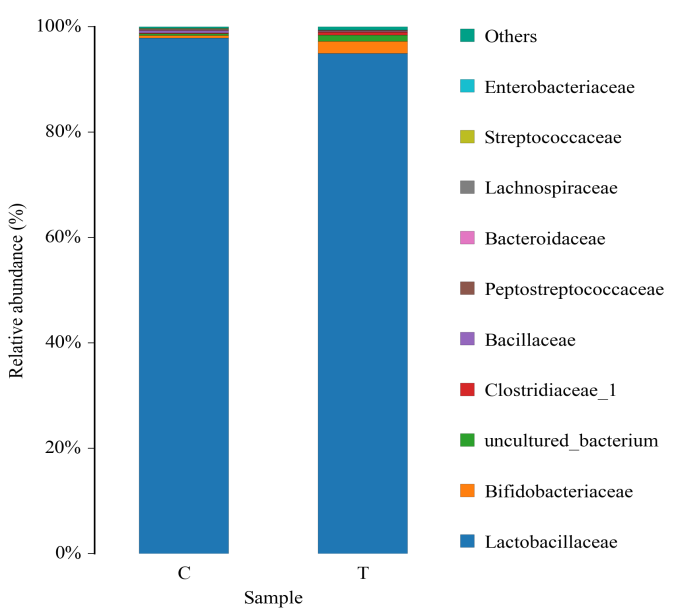 |
| E. At genus level |  |
| 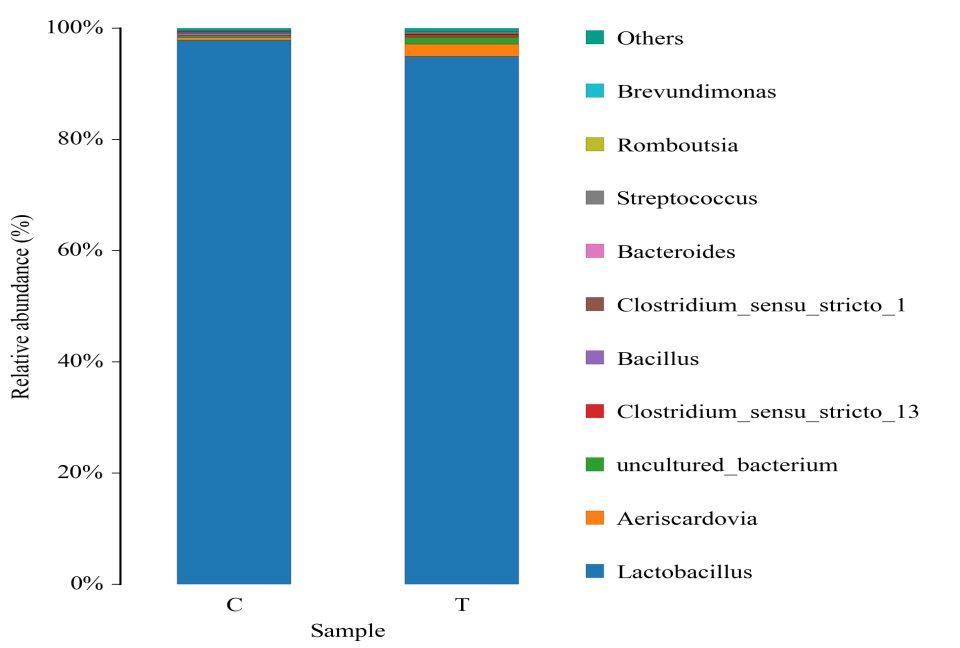 | |

**Supplementary Figure 1.** Composition of gut microbiota at various taxonomic levels of layers


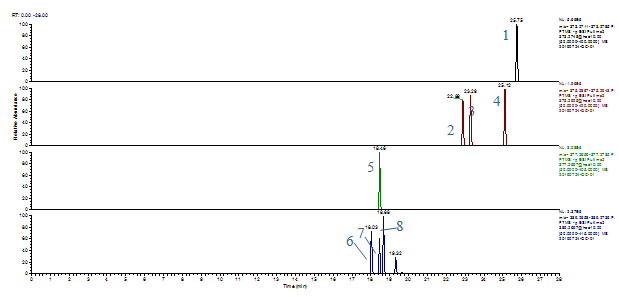

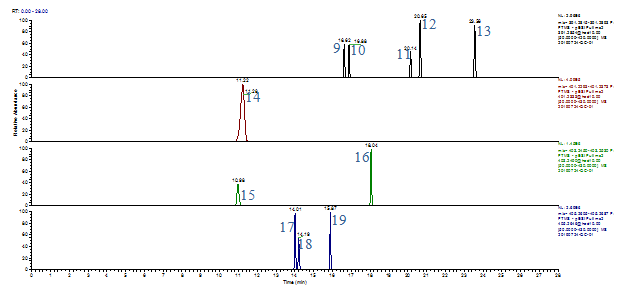


**
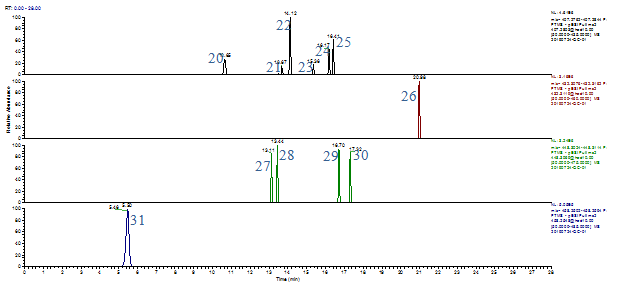

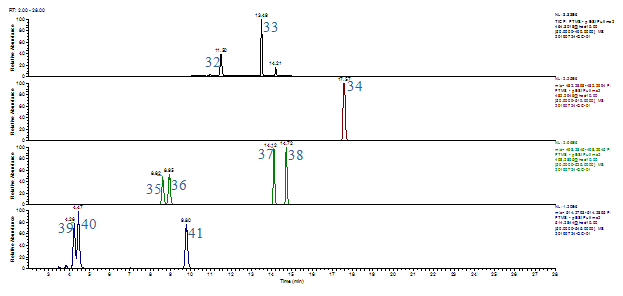
**

**Supplementary Figure 2.** Ion recording chromatograms of bile acid standard solutions. (1) DHLCA (dehydrolithocholic acid); (2) ALCA (allolithocholic acid); (3) ILCA (isolithocholic acid); (4 LCA (lithocholic acid); (5) 23-NDCA (23-Nordeoxycholic acid); (6) 7-KLCA (7-ketolithocholic acid); (7) 12-KLCA (12-ketolithocholic acid); (8) ApCA (apocholic acid); (9) UDCA (ursodeoxycholic acid); (10) HDCA (hyodeoxycholic acid); (11) CDCA (chenodeoxycholic acid); (12) DCA (deoxycholic acid); (13) IDCA (isodeoxycholic acid); (14) DHCA (dehydrocholic acid); (15) 7,12-DKLCA (7,12-diketolithocholic acid); (16) 6,7-DKLCA (6,7-diketolithocholic acid); (17) 7-KDCA (7-ketodeoxycholic acid); (18) 12-DHCA (12-dehydrocholic acid); (19) 3-DHCA (3-dehydrocholic acid); (20) UCA (ursocholic acid); (21) αMCA (α-muricholic acid); (22) βMCA (β-muricholic acid); (23) λMCA (λ-muricholic acid); (24) AlCA (allocholic acid); (25) CA (cholic acid); (26) GLCA (glycolithocholic acid); (27) GUDCA (glycoursodeoxycholic acid); (28) GHDCA (glycohyodeoxycholic acid); (29) GCDCA (glycochenodeoxycholic acid); (30) GDCA (glycodeoxycholic acid); (31) GDHCA (glycodehydrocholic acid); (32) GλMCA (glyco-λ-muricholic acid); (33) GCA (glycocholic acid); (34) TLCA (taurolithocholic acid); (35) TUDCA (tauroursodeoxycholic acid); (36) THDCA (taurohyodeoxycholic acid); (37) TCDCA (taurochenodeoxycholic acid); (38) TDCA (taurodeoxycholic acid); (39) TαMCA (tauro α-muricholic acid); (40) TβMCA (tauro β-muricholic acid); (41) TCA (taurocholic acid).
